# Utility of intracranial EEG networks depends on re-referencing and connectivity choice

**DOI:** 10.1093/braincomms/fcae165

**Published:** 2024-05-13

**Authors:** Haoer Shi, Akash Ranjan Pattnaik, Carlos Aguila, Alfredo Lucas, Nishant Sinha, Brian Prager, Marissa Mojena, Ryan Gallagher, Alexandra Parashos, Leonardo Bonilha, Ezequiel Gleichgerrcht, Kathryn A Davis, Brian Litt, Erin C Conrad

**Affiliations:** Department of Bioengineering, School of Engineering and Applied Sciences, University of Pennsylvania, Philadelphia, PA 19104, USA; Center for Neuroengineering and Therapeutics, University of Pennsylvania, Philadelphia, PA 19104, USA; Department of Bioengineering, School of Engineering and Applied Sciences, University of Pennsylvania, Philadelphia, PA 19104, USA; Center for Neuroengineering and Therapeutics, University of Pennsylvania, Philadelphia, PA 19104, USA; Department of Bioengineering, School of Engineering and Applied Sciences, University of Pennsylvania, Philadelphia, PA 19104, USA; Center for Neuroengineering and Therapeutics, University of Pennsylvania, Philadelphia, PA 19104, USA; Department of Bioengineering, School of Engineering and Applied Sciences, University of Pennsylvania, Philadelphia, PA 19104, USA; Center for Neuroengineering and Therapeutics, University of Pennsylvania, Philadelphia, PA 19104, USA; Center for Neuroengineering and Therapeutics, University of Pennsylvania, Philadelphia, PA 19104, USA; Department of Neurology, Perelman School of Medicine, University of Pennsylvania, Philadelphia, PA 19104, USA; Center for Neuroengineering and Therapeutics, University of Pennsylvania, Philadelphia, PA 19104, USA; Center for Neuroengineering and Therapeutics, University of Pennsylvania, Philadelphia, PA 19104, USA; Center for Neuroengineering and Therapeutics, University of Pennsylvania, Philadelphia, PA 19104, USA; Department of Neurology, Medical University of South Carolina, Charleston, SC 29425, USA; Department of Neurology, Emory University, Atlanta, GA 30325, USA; Department of Neurology, Emory University, Atlanta, GA 30325, USA; Center for Neuroengineering and Therapeutics, University of Pennsylvania, Philadelphia, PA 19104, USA; Department of Neurology, Perelman School of Medicine, University of Pennsylvania, Philadelphia, PA 19104, USA; Department of Bioengineering, School of Engineering and Applied Sciences, University of Pennsylvania, Philadelphia, PA 19104, USA; Center for Neuroengineering and Therapeutics, University of Pennsylvania, Philadelphia, PA 19104, USA; Department of Neurology, Perelman School of Medicine, University of Pennsylvania, Philadelphia, PA 19104, USA; Center for Neuroengineering and Therapeutics, University of Pennsylvania, Philadelphia, PA 19104, USA; Department of Neurology, Perelman School of Medicine, University of Pennsylvania, Philadelphia, PA 19104, USA

**Keywords:** intracranial EEG, pre-processing, connectivity, relative entropy

## Abstract

Studies of intracranial EEG networks have been used to reveal seizure generators in patients with drug-resistant epilepsy. Intracranial EEG is implanted to capture the epileptic network, the collection of brain tissue that forms a substrate for seizures to start and spread. Interictal intracranial EEG measures brain activity at baseline, and networks computed during this state can reveal aberrant brain tissue without requiring seizure recordings. Intracranial EEG network analyses require choosing a reference and applying statistical measures of functional connectivity. Approaches to these technical choices vary widely across studies, and the impact of these technical choices on downstream analyses is poorly understood. Our objective was to examine the effects of different re-referencing and connectivity approaches on connectivity results and on the ability to lateralize the seizure onset zone in patients with drug-resistant epilepsy. We applied 48 pre-processing pipelines to a cohort of 125 patients with drug-resistant epilepsy recorded with interictal intracranial EEG across two epilepsy centres to generate intracranial EEG functional connectivity networks. Twenty-four functional connectivity measures across time and frequency domains were applied in combination with common average re-referencing or bipolar re-referencing. We applied an unsupervised clustering algorithm to identify groups of pre-processing pipelines. We subjected each pre-processing approach to three quality tests: (i) the introduction of spurious correlations; (ii) robustness to incomplete spatial sampling; and (iii) the ability to lateralize the clinician-defined seizure onset zone. Three groups of similar pre-processing pipelines emerged: common average re-referencing pipelines, bipolar re-referencing pipelines and relative entropy-based connectivity pipelines. Relative entropy and common average re-referencing networks were more robust to incomplete electrode sampling than bipolar re-referencing and other connectivity methods (Friedman test, Dunn–Šidák test *P* < 0.0001). Bipolar re-referencing reduced spurious correlations at non-adjacent channels better than common average re-referencing (Δ mean from machine ref = −0.36 versus −0.22) and worse in adjacent channels (Δ mean from machine ref = −0.14 versus −0.40). Relative entropy-based network measures lateralized the seizure onset hemisphere better than other measures in patients with temporal lobe epilepsy (Benjamini–Hochberg-corrected *P* < 0.05, Cohen’s *d*: 0.60–0.76). Finally, we present an interface where users can rapidly evaluate intracranial EEG pre-processing choices to select the optimal pre-processing methods tailored to specific research questions. The choice of pre-processing methods affects downstream network analyses. Choosing a single method among highly correlated approaches can reduce redundancy in processing. Relative entropy outperforms other connectivity methods in multiple quality tests. We present a method and interface for researchers to optimize their pre-processing methods for deriving intracranial EEG brain networks.

## Teaser

Shi *et al*. report that the choice of re-referencing and functional connectivity measure alters the interpretation of the intracranial EEG epileptic network in a multi-centre study of 125 subjects. The authors evaluate methods’ similarity, robustness to spatial sampling, spurious correlations and performance on seizure onset hemisphere lateralization.

## Graphical Abstract

**Graphical Abstract:**
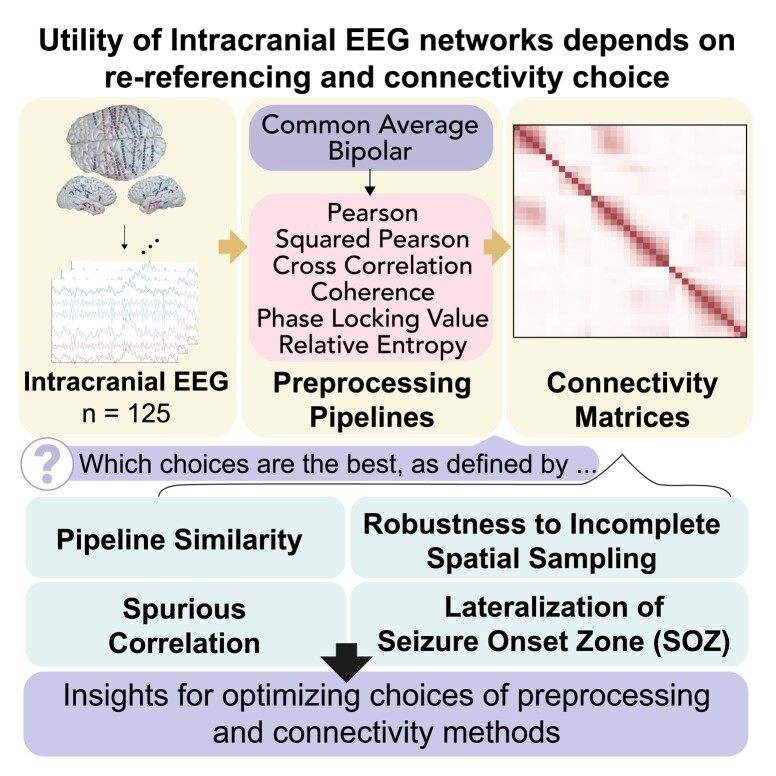

## Introduction

Intracranial EEG (iEEG) recordings give unprecedented access to brain activity. The high temporal resolution and precise spatial resolution of iEEG enable localization of seizures and epileptic networks. Recent research focuses on intracranial ‘functional connectivity’ (FC), the statistical association between sources of brain activity. iEEG FC analysis has probed both normal and abnormal brain function^1-9^ and relies on defining a measure of association between brain sources. How this association is defined varies widely across studies, using different approaches to handle the time- and frequency-dependent characteristics of iEEG signals. Studies also use various approaches to re-reference EEG signals to eliminate the influence of a common recording reference prior to measuring connectivity. These choices affect downstream connectivity measurements and analyses.^10^ How different choices of functional connectome generation and references affect the seizure localization results from iEEG connectivity analyses is poorly understood. This knowledge gap limits the interpretation and reproducibility of iEEG FC studies.

Here we evaluate the effect of pre-processing and FC on interictal resting-state brain network analysis in a cohort of patients with drug-resistant epilepsy. We choose combinations of 2 different re-referencing methods and 24 commonly used connectivity measures and evaluate their similarity, robustness to spatial sampling and the introduction of spurious correlations. To understand the clinical impact of pre-processing choices, we determined how well these measures lateralize the clinician-defined seizure onset zone (SOZ). Our findings offer valuable insights into method selection for future iEEG research and help improve their standardization.

## Materials and methods

### Patient information

iEEG and imaging data collection for research was approved by the Institutional Review Boards of the Hospital of the University of Pennsylvania (HUP) and Medical University of South Carolina (MUSC). Inclusion criteria for this study included (i) pre-implant and post-implant MRI and post-implant CT scans for electrode localization and (ii) iEEG recordings. For lateralizing the SOZ, additional inclusion criteria included (iii) concordant SOZ lateralization from the pre-implantation surgical conference for ground truth labels and (iv) bilateral depth coverage of hippocampus and amygdala. For similarity and robustness analysis, 107 patients with grey or white matter origin electrodes were included. In the HUP cohort, 35 patients were implanted with electrocorticography (ECoG) grids, strips and depths, and 72 patients were implanted with stereoelectroencephalography (SEEG) depths only. iEEG recordings from an additional 18 patients from Medical University of South Carolina (MUSC) were included as part of the validation analysis, and all implantations were SEEG depths. Further information about the electrode configurations is included in the Supplementary material. SOZ lateralization was conducted on patients with a unilateral SOZ in the temporal lobe, identified during clinical evaluation and included 30 HUP patients and 16 MUSC patients. A description of patients in both cohorts is presented in Table 1. Detailed processing procedures including data recording, SOZ lateralization and electrode localization are included in the Supplementary material.

**Table 1:** Clinical and recording information of the HUP and MUSC data set

	HUP data set		MUSC data set	
	Similarity analysis sample (*N* = 107)	SOZ analysis sample (*N* = 30)	Similarity analysis sample (*N* = 18)	SOZ analysis sample (*N* = 16)
Gender				
Male, *N* (%)	47 (43.9)	11 (36.7)	8 (44.4)	8 (50.0)
Female, *N* (%)	55 (51.4)	17 (56.7)	9 (50.0)	7 (43.8)
Age at onset in years, median (range)	15.0 (0.0–52.0)	1 (1.0–17.0)	25.0 (8.0–59.0)	23.0 (8.0–59.0)
Age at implant in years, median (range)	36.0 (16.0–62.0)	37.0 (22.0–69.0)	38.0 (23.0–73.0)	35 (23.0–63.0)
SOZ laterality, *N* (%)				
Left	53 (49.5)	20 (66.7)	5 (27.8)	5 (31.2)
Right	32 (29.9)	10 (33.3)	9 (50.0)	11 (68.8)
Bilateral	22 (20.6)	0 (0.0)	3 (16.7)	0 (0.0)
SOZ localization, *N* (%)				
Temporal	62 (57.9)	30 (100.0)	17 (94.4)	16 (100.0)
Other	43 (40.2)	0 (0.0)	0 (0.0)	0 (0.0)
Unknown	2 (1.9)	0 (0.0)	1 (5.6)	0 (0.0)
Number of contacts, median (range)	92 (33–191)	36 (8–72)	73 (8–192)	60 (20–60)
Implant type, *N* (%)				
ECoG + depths	35 (32.7)	8 (26.7)		
SEEG	72 (67.2)	22 (67.3)		
Sampling rate, *N* (%)				
256 Hz	2 (1.87)	1 (3.3)	0 (0.0)	0 (0.0)
500 Hz	18 (16.82)	6 (20.0)	0 (0.0)	0 (0.0)
512 Hz	59 (55.14)	12 (40.0)	6 (33.3)	5 (31.2)
1024 Hz	27 (25.23)	11 (36.7)	0 (0.0)	0 (0.0)
2048 Hz	1 (0.93)	0 (0.0)	12 (66.7)	11 (68.8)

SOZ, seizure onset zone; ECoG, electrocorticography; SEEG, stereoelectroencephalography.

### iEEG data collection

**Figure 1:**
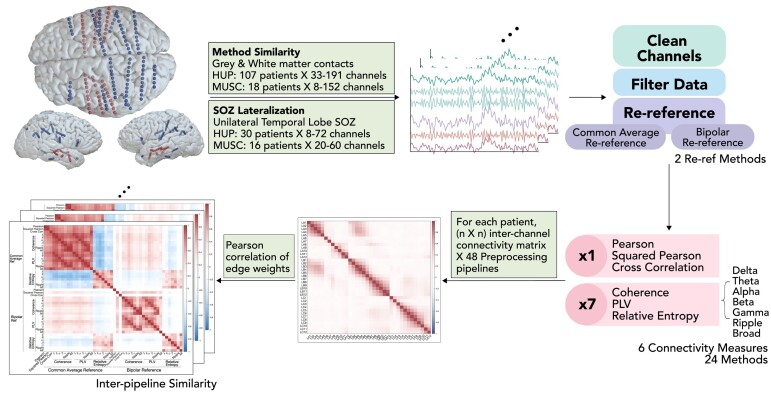
**Toolkit and analysis pipeline**. Intracranial EEG electrodes are implanted to record epileptic networks. We apply an open-access tool to clean signals, filter the data and apply 48 pre-processing pipelines, combinations of re-referencing and connectivity methods. This approach is applied to 125 patients across two epilepsy centres. HUP, Hospital of the University of Pennsylvania; MUSC, Medical University of South Carolina; SOZ, seizure onset zone; PLV, phase locking value.

iEEG data were recorded using ECoG and SEEG at 256–2048 Hz (Table 1). Electrodes were localized on brain imaging using the iEEG-recon pipeline^11^ and assigned to grey or white matter tissues or cerebrospinal fluid locations (Fig. 1). Contacts localized to cerebrospinal fluid location were excluded from analysis. Data were downloaded from iEEG.org,^12^ an open-source cloud repository of research and clinical EEG data. Five random 2-min interictal data epochs sampled between 1pm and 3pm were selected for each patient, chosen to be long enough to observe stable functional networks^13^ and to maximize the likelihood that patients were awake. Awake clips were chosen to standardize data across patients and to minimize variability in brain activity across different sleep stages.^14^ Data were filtered using a fourth-order infinite impulse response notch filter at 60 Hz to remove power line noise. Channels with overwhelming noise were discarded. Specific thresholds for identifying noise are available elsewhere.^15^

### Re-referencing methods

Data were originally referenced to a distant electrode with minimal activity, selected by the clinical team. To eliminate the effect of the original referencing on the recorded data, two re-referencing methods were applied as part of the pre-processing pipeline: common average referencing (CAR) and bipolar referencing (BR). For each data epoch, we applied CAR by calculating the mean of all voltage values at a single time point and subtracting this value from all artefact-free channels localized to grey or white matter tissue.^16^ We applied BR by subtracting voltage values in pairs of contacts that were closest to each other by electrode name (i.e. LA01, LA02 and LA03 were transformed to LA01–LA02 and LA02–LA03). Bipolar pairs were only considered within one electrode (i.e. all pairs within depth electrode LA only). To minimize the amount of lost data for contacts that contained artefact, we preserved skipped connection pairs (i.e. LA01–LA03 was allowed when LA02 was missing).

### Connectivity methods

To construct FC networks from iEEG data, we applied both time and spectral domain connectivity methods between each pair of channels in each epoch. We chose methods commonly reported in the iEEG FC literature, and we describe each method’s conceptual and mathematical definitions in Supplementary Table 1. Time domain connectivity methods include Pearson correlation, squared Pearson correlation and cross-correlation with a maximum time lag of 200 ms. Spectral domain connectivity methods included magnitude-squared coherence and phase locking value (PLV). Finally, we implemented relative entropy (RE), an amplitude-based connectivity method^17^ that computes differences in the probability distribution of two signals’ amplitudes. All connectivity measures were calculated within 2-s windows to approximate stationarity of the pairs of signals^18,19^ and averaged across each epoch. Coherence, PLV and RE were calculated over seven canonical frequency bands: delta (0.5–4 Hz), theta (4–8 Hz), alpha (8–12 Hz), beta (12–30 Hz), gamma (30–80 Hz), ripple (80–250 Hz) and broadband (0.5–250 Hz). Since RE is not a symmetric measure (i.e. RE of LA01 to LA02 does not equal RE of LA02 to LA01), we applied the formula in both directions and chose the higher value to represent this connectivity method. This resulted in 24 connectivity methods and 48 combinations of re-referencing and connectivity analysis (Fig. 1), henceforth referred to as ‘pre-processing pipelines’. Applying each pre-processing pipeline resulted in a ‘FC network’, where ‘nodes’ represent each channel and ‘edges’ represent the pairwise connectivity between nodes.

### Similarity in networks derived from different pre-processing pipelines

We first compared the similarity between networks derived from the different pre-processing pipelines (Fig. 1). For each patient, we applied all 48 pre-processing pipelines. To determine how similar each network representation was to each other, we extracted all edges from each FC network and calculated the Pearson correlation between edge weights across FC networks. This yielded a 2D similarity matrix where rows and columns represented each pre-processing pipeline and element values closer to 1 (0) indicated more similar (different) FC networks. Across patients, we averaged the matrix of similarity values to determine group-level similarity values. We also calculated the similarity between node-level measurements derived from different pre-processing pipelines (see Supplementary material). We identified clusters in pre-processing pipelines by using hierarchical clustering. The R package bootcluster,^20^ with 20 and 1000 bootstrapping resamples, was used to determine the number of clusters and assessment of stability (range 0–1), respectively. Whilst conventional methods such as the elbow method favour choosing two clusters, three clusters were chosen to further distinguish pipelines of different re-referencing methods. This analysis was applied independently on the HUP and MUSC data sets, and cluster consistency was evaluated using a variety of clustering algorithms and statistical tests for robustness (see Supplementary material). For visualization, we performed dimensionality reduction on the similarity values between pre-processing pipelines to two dimensions using *t*-distributed stochastic neighbour embedding (*t*-SNE).

### Spurious correlation

The presence of a common reference between neural sources can introduce false correlations between uncoupled sources.^10^ To compare the introduction of spurious correlations across different re-referencing methods, we simulated iEEG data using the Fieldtrip ‘connectivitysimulation’ function^21^ and replicated previously published methods for simulating spurious correlations.^10^ Briefly, data from eight uncorrelated or correlated channels were simulated for 100 trials at 512 Hz sampling rate and 2-s trial length. This simulator linearly mixed eight unobserved signals for each of the eight observed signals. A common signal source was applied to all channels as a simulation of the machine reference on which iEEG data are recorded. Whilst uncorrelated data were used to reveal the introduction of correlations, correlated data were more representative of the realistic situation in which signals from adjacent contacts were more correlated with each other.^1,22^ The channels were considered to be eight consecutive contacts on a single depth electrode, with correlations across channels estimated according to the exponential of the negative Euclidean distance.^23^ Each pre-processing pipeline was applied to the simulated data. To further compare the effects of re-referencing, the connectivity between adjacent (directly next to each other on the same electrode, e.g. channels LA01 and LA02) and non-adjacent (not directly next to each other on the same electrode, e.g. channels LA01 and LA03) contacts was extracted and averaged, respectively.

### Robustness to electrode spatial sampling

iEEG analysis is limited by where clinicians choose to place electrodes in the brain. Our recent work suggests that different graph theory measures have variable sensitivity to choice of electrode placement, but the sensitivity of different connectivity measures and reference choices to electrode placement is unknown.^24^ We investigated the sensitivity of different pre-processing pipelines to changes in electrode spatial sampling. We adapted a metric for the reliability of each pre-processing pipeline from previous literature.^24,25^ Briefly, for each FC network, a portion of channels was randomly removed and the corresponding node strength was calculated. Channels were randomly subsampled at 20, 40, 60 and 80% of the original sample size at the patient level, and this procedure was repeated over 1000 iterations per removal percentage. Reliability of the method was calculated according to previous work.^24,25^ Reliability *R* is defined as the true variance *σ*_T_ divided by the total variance *σ*_X_, where *σ*_X_ = *σ*_T_ + *σ*_E_, the variance of the error. Specifically, *σ*_E_ is the variance of the metric across the 1000 iterations, averaged across iEEG contacts, whilst *σ*_T_ is the metric variance across iEEG contacts averaged over all iterations.

### SOZ lateralization

To investigate how well each pipeline lateralizes the SOZ, we first restricted the analysis to patients with a unilateral SOZ in the temporal lobe, given that (i) temporal lobe epilepsy is the most common localization of drug-resistant epilepsy in adults^26^ and (ii) the temporal lobes are commonly sampled bilaterally at the two centres, facilitating side-to-side comparisons for laterality analysis. We hypothesized that the SOZ hemisphere would demonstrate lower FC than the non-SOZ hemisphere, given that both seizure onset and non-seizure onset channels are sampled within the SOZ hemisphere. We then selected a cohort of patients that had up to three lateral SEEG depth electrodes implanted in each hemisphere, which recorded from the amygdala and hippocampus, common surgical targets in temporal lobe epilepsy. We identified the lateral depth electrodes by visual inspection and used all contacts that passed artefact rejection for this analysis. Each electrode consists of up to 12 contacts, resulting in up to 72 channels of data (Fig. 1). Given the focus on epilepsy lateralization, only contacts with symmetric counterparts in the contralateral hemisphere were included in downstream analysis (e.g. LA01 and RA01). We performed this spatial downsampling of electrodes in order to control for the difference in FC in different anatomical locations^6,7^ as well as for the spatial sampling bias wherein clinicians tend to place electrodes more densely around the suspected SOZ, leading towards higher connectivity measurements in the SOZ.^18,27^ To compare between the side of the clinician-defined SOZ and the contralateral side, the connectivity in each temporal lobe was averaged.

### Statistical analysis

All statistical analyses were performed using MATLAB (MathWorks, Inc., 2021b). A two-sided *P* < 0.05 was considered significant. We used non-parametric Wilcoxon signed-rank tests and Friedman tests to examine differences in robustness across re-referencing and connectivity methods, respectively. *Post hoc* Dunn–Šidák multiple comparisons tests were further conducted for the Friedman test to identify significantly different method pairs. A paired *t*-test with Benjamini–Hochberg correction to correct for testing multiple pre-processing pipelines was performed for the SOZ versus non-SOZ side connectivity comparison. Effect sizes were quantified using Cohen’s *d*, where values of 0.20, 0.50 and 0.80 were considered as small, medium and large effects, respectively.

## Results

### Patient and electrode information

A total of 107 and 18 patients, respectively, were included in the analysis of the HUP and MUSC data sets after screening for complete imaging, iEEG and demographic data. We applied electrode localization to grey and white matter and discarded channels localized to the cerebrospinal fluid and those with overwhelming artefacts. Since the location of iEEG implantation is solely determined by clinical need, the number of electrode contacts that met localization and artefact criteria varied from 33 to 191 contacts across patients for the HUP data set and from 8 to 152 contacts for the MUSC data set. Contacts in both grey and white matter were included in subsequent analyses. Extended information on patient demographics are given in Table 1.

### Unsupervised clustering reveals similar connectivity and re-referencing pipelines

**Figure 2:**
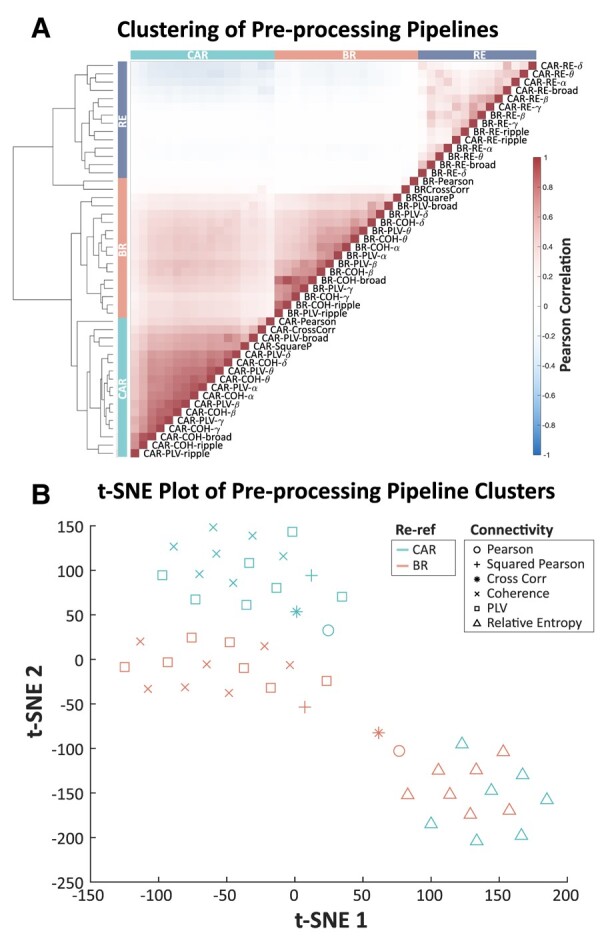
**Clustering of different pre-processing pipelines**. (**A**) Each row and column represents a pre-processing pipeline, the three categories along the axes represent three major clusters: common average re-referencing cluster, bipolar re-referencing cluster and RE cluster. Hierarchical clustering was applied to the networks, and the resulting dendrogram is shown on the left axis. Cells show the Pearson correlation coefficient between pairs of pre-processing pipelines. Pre-processing pipelines are indicated as [re-referencing method]-[connectivity method]-[frequency band (where applicable)]. (**B**) The similarity matrix was further reduced to two dimensions using *t*-SNE. Each data point refers to a pre-processing pipeline. CAR, common average re-referencing; BR, bipolar re-referencing; RE, relative entropy; Pearson, Pearson correlation; CrossCorr, cross-correlation; SquareP, squared Pearson correlation; PLV, phase locking value; COH, magnitude-squared coherence. Greek letters refer to canonical frequency bands.

We calculated FC networks with 48 combinations of re-referencing and connectivity pre-processing pipelines. Hierarchical clustering revealed three major clusters (Fig. 2A): RE pipelines, BR pipelines and CAR pipelines (average clustering stability = 0.86). RE formed a unique cluster that included all frequency bands and re-referencing methods. For all other connectivity measures, pre-processing pipelines clustered more strongly by re-referencing methods; CAR and BR pipelines formed the other two major clusters. The *t*-SNE plot similarly exhibited these three major clusters (Fig. 2B). Within the CAR or BR cluster, coherence and PLV were highly similar in that coherence and PLV of the same band were clustered together, indicating that frequency band effects were dominant. Pipelines using CAR were more intercorrelated, suggesting that the choice of connectivity became less influential when using CAR. We observed consistent results following replication with different FC network aggregation methods, clustering algorithms and data from a second epilepsy centre (Supplementary Fig. 1). To ensure that our results were not biased towards using a signed Pearson correlation that isolated a RE cluster, we extended our analysis in two ways: (i) we re-computed similarity using the absolute value of RE and (ii) we transformed RE into all positive values by applying $\frac{1}{1\;+\;x}$, where *x* is a calculated value of RE (Supplementary Fig. 2).

### Common average re-referencing and RE are robust to spatial sampling

**Figure 3:**
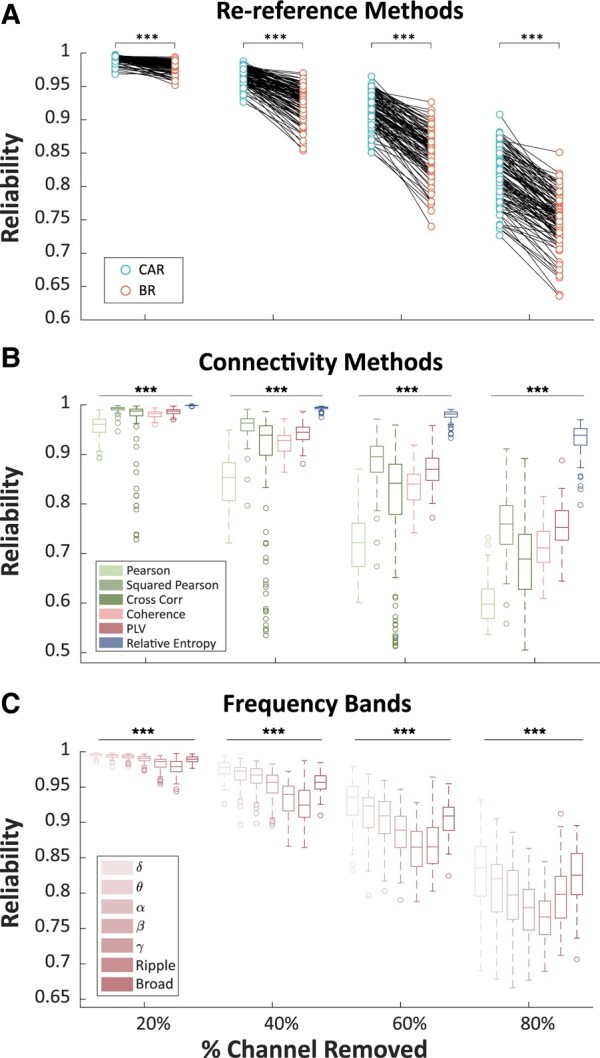
**Robustness of spatial sampling of different pre-processing pipelines**. (**A**) The reliability of different pipelines involving the focussed re-referencing method was averaged. Each point represents a patient. A Wilcoxon signed-rank test was applied. 20%: Wilcoxon signed-rank test *Z* = 8.90, *P* < 0.001; 40%: *Z* = 8.93, *P* < 0.001; 60%: *Z* = 8.92, *P* < 0.001; 80%: *Z* = 8.92, *P* < 0.001. (**B**) Average reliability of the connectivity method across different re-referencing methods was reported. Non-parametric Friedman tests were used to examine differences in robustness across methods. 20%: Friedman test *χ*^2^ = 425.21, *P* < 0.001; 40%: *χ*^2^ = 430.33, *P* < 0.001; 60%: *χ*^2^ = 425.42, *P* < 0.001; 80%: *χ*^2^ = 426.78, *P* < 0.001. *Post hoc* Dunn–Šidák multiple comparisons tests were further conducted for the Friedman test to identify significantly different method pairs. (**C**) Boxplots group each spectral connectivity method by the canonical frequency band that was used. Friedman test and multiple comparisons corrections were applied. 20%: Friedman test *χ*^2^ = 443.04, *P* < 0.001; 40%: *χ*^2^ = 426.61, *P* < 0.001; 60%: *χ*^2^ = 384.29, *P* < 0.001; 80%: *χ*^2^ = 304.33, *P* < 0.001. CAR, common average re-referencing; BR, bipolar re-referencing; RE, relative entropy; Pearson, Pearson correlation; CrossCorr, cross-correlation; SquareP, squared Pearson correlation; PLV, phase locking value; COH, magnitude-squared coherence. Greek letters refer to canonical frequency bands. *** represents *P* < 0.001.

The reliability of all pre-processing pipelines decreased as more channels were removed (Fig. 3). The mean reliability of CAR was significantly higher than that of BR at all four subsampling percentages: 20, 40, 60 and 80% (signed-rank test: *P* < 0.001). For connectivity measures, the mean reliability was significantly different at 20% (Friedman test: *χ*^2^ = 425.21, *P* < 0.001), 40% (*χ*^2^ = 430.33, *P* < 0.001), 60% (*χ*^2^ = 425.42, *P* < 0.001) and 80% (*χ*^2^ = 426.78, *P* < 0.001) random channel removal. Specifically, RE was significantly more reliable than all other connectivity measures at all percentages (*post hoc* Dunn–Šidák test: *P* < 0.001 for all percentages), whilst squared Pearson ranked second. We validated these findings using the MUSC data set, where we observed similar trends (Supplementary Fig. 3). The reliability of networks measuring specific frequency bands gradually decreased from the delta band to the ripple band. Detailed statistics are summarized in Supplementary Tables 2 and 3.

### Unsupervised clusters and robustness to spatial sampling is preserved after thresholding the number of channels

As the number of iEEG channels in the HUP and MUSC data set ranged from 8 to 191, the interpretation of the FC network could vary dramatically. As a result, we repeated our analyses for clusters of pre-processing pipelines and robustness of spatial sampling on three subsets of patients based on the number of channels recorded. We tested three cut-offs: 60, 73 and 92 channels, which roughly correspond to the 20th, 30th and 50th percentiles of the distribution of channels across patients. For all three cut-offs, we were able to identify three major clusters: RE pipelines, BR pipelines and CAR pipelines. CAR demonstrated higher reliability to spatial sampling than BR at all three cut-offs (all percentages: signed-rank test: *P* < 0.001). The mean reliability of connectivity measures differed at all three cut-offs (all percentages: Friedman’s test: *P* < 0.001), with RE showing the highest reliability (*post hoc* Dunn–Šidák test: *P* < 0.01 for all percentages). As the findings from all three cut-offs are consistent, figures from the 92-channel cut-off are presented (Supplementary Fig. 6).

### Re-referencing has different effects on adjacent and non-adjacent connectivity pairs

**Figure 4:**
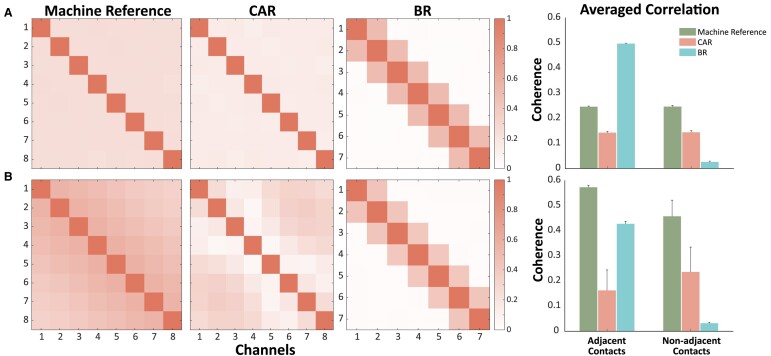
**Robustness against spurious correlation of different re-referencing methods**. (**A**) On simulated uncorrelated data. (**B**) On simulated correlated data. A paired samples *t*-test was conducted for each pair of re-referencing methods. (**A**) Adjacent contacts: machine reference versus CAR, *t*(6) = 31.4, *P* < 0.001; machine reference versus BR, *t*(5) = −113.4, *P* < 0.001; CAR versus BR, *t*(5) = −172.5, *P* < 0.001. Non-adjacent contacts: machine reference versus CAR, *t*(20) = 86.7, *P* < 0.001; machine reference versus BR, *t*(14) = 179.8, *P* < 0.001; CAR versus BR, *t*(14) = 119.9, *P* < 0.001. (**B**) Adjacent contacts: machine reference versus CAR, *t*(6) = 11.8, *P* < 0.001; machine reference versus BR, *t*(5) = 24.6, *P* < 0.001; CAR versus BR, *t*(5) = −9.6, *P* < 0.001. Non-adjacent contacts: machine reference versus CAR, *t*(20) = 6.3, *P* < 0.001; machine reference versus BR, *t*(14) = 26.9, *P* < 0.001; CAR versus BR, *t*(14) = 7.6, *P* < 0.001. Left, broadband coherence matrix on machine-reference, common-average-reference or bipolar-reference data. Right, average coherence for adjacent contacts or non-adjacent contacts. CAR, common average re-referencing; BR, bipolar re-referencing.

Both re-referencing methods reduced spurious correlation introduced through machine reference. Nevertheless, CAR and BR showed different patterns in adjacent and non-adjacent channels (Fig. 4). After fixing coherence as the connectivity method and applying a simulation of uncorrelated data (Fig. 4A), using CAR resulted in lower spurious correlations compared with the machine reference [machine reference: mean (SD) = 0.25 (0.004), CAR: mean (SD) = 0.14 (0.005), paired *t*-test: *P* < 0.001]. BR outperformed CAR at non-adjacent channels, achieving a coherence of 0.027, though it performed worse at adjacent channels (mean coherence = 0.498). Coherence was greater at adjacent channels than at non-adjacent channels (mean: 0.56 versus 0.45) when recording using the machine reference for correlated data (Fig. 4B) due to the introduction of distance-based correlation; pairs of contacts that were more proximal on the electrode were simulated to have a higher connectivity than pairs that were distal. Applying CAR resulted in higher coherence at non-adjacent channels (mean = 0.23) than adjacent channels (mean = 0.16). Similarly, BR significantly reduced connectivity at non-adjacent channels (mean = 0.09) compared with adjacent channels (mean = 0.42).

### RE outperforms other connectivity measures in epilepsy lateralization

**Figure 5:**
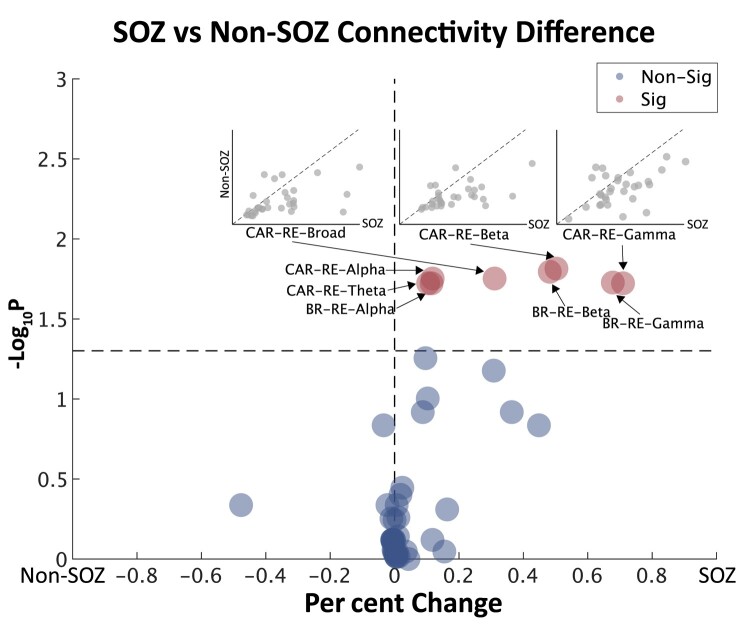
**SOZ lateralization performances of different pre-processing pipelines**. Outer: SOZ versus non-SOZ side global connectivity differences. Horizontal axis represents SOZ versus non-SOZ global connectivity change. Vertical axis indicates Benjamini–Hochberg-corrected paired *t-*test significance of SOZ versus non-SOZ differences. Each dot represents a pre-processing pipeline. A positive per cent change indicates higher connectivity in the SOZ hemisphere. Inner: embedded are the sample scatter plots of SOZ versus non-SOZ side global connectivity for pipelines showing significant differences (CAR-RE-Broad, *t* = 3.513, *P* = 0.018; CAR-RE-Beta, *t* = 4.083, *P* = 0.015; CAR-RE-Gamma, *t* = 3.264, *P* = 0.019). The *P*-values and corresponding *t*-statistics of other pipelines are available in Supplementary Table 4. The horizontal and vertical axes represent the SOZ and non-SOZ side global connectivity, respectively. Each dot in the embedded plots represents a single patient. The dashed line indicates equal connectivity at both sides. CAR, common average re-referencing; BR, bipolar re-referencing; RE, relative entropy; Sig, statistically significant following multiple comparisons correction.

We next introduced each individual’s epilepsy lateralization to validate the previous technical findings in a clinical application. FC networks computed using RE in the alpha, beta and gamma bands with either re-referencing method and in theta and broad bands with CAR exhibited significantly higher connectivity in the seizure onset hemisphere than the non-seizure onset hemisphere, with medium to large effect sizes (Fig. 5, Benjamini–Hochberg-corrected *P* < 0.05, Cohen's *d*: 0.60–0.76; see Supplementary Table 4 for detailed statistics). No other connectivity measures exhibited significant SOZ versus non-SOZ hemisphere differences. Validation on the MUSC data set revealed no significant differences between SOZ and non-SOZ hemispheres after correcting for multiple comparisons, though the smaller sample size from the external validation site may have resulted in an underpowered statistical test. RE-based pipelines in the MUSC cohort trended towards statistical significance in the SOZ versus non-SOZ side connectivity comparison (Supplementary Fig. 4), with comparable per cent changes and effect sizes to that observed in the HUP data set (Supplementary Table 4). We additionally computed the number of interictal spikes in each epoch using a validated spike detector and found the number of spikes was significantly higher in the SOZ hemisphere than the non-seizure onset hemisphere (Supplementary Fig. 7A). To further investigate the association between RE and interictal spikes, we visualized the occurrence of interictal spikes on iEEG traces from low, medium and high RE values in an example patient (Supplementary Fig. 5). We found that high RE values occurred in 2-s windows when interictal spikes were present in one of the two channels.

### An open-source cross-platform user interface for iEEG pre-processing

To promote open-access code and encourage others to replicate our findings, we built an interactive graphical user interface (GUI) for our methods in both MATLAB and Python platforms. The GUI supports artefact rejection, filtering, re-referencing and additional processing such as feature extraction and connectivity analysis. To encourage the development of additional tools, the interface is designed in a modular format. A codeless interface enabled through MATLAB live script allows simple and rapid pre-processing and testing. The GUI is available at the following links: Python (github.com/penn-cnt/CNT_Preprocessing_Toolkit/blob/main/python/pipeline_demo.ipynb) and MATLAB (github.com/penn-cnt/CNT_Preprocessing_Toolkit/blob/main/matlab/pipeline_demo.mlx).

## Discussion

The study of functional networks in brain activity measured with intracranial electrophysiology, such as EEG, has implications for understanding the physiology of a number of neurological and psychiatric disorders including Parkinson’s disease,^28^ schizophrenia,^29,30^ epilepsy^31^ and depression.^32^ We pursued this study because it is unknown how the choice of processing methods alters the interpretation of network analyses. To answer this question, we evaluated commonly used re-referencing and FC methods in network analyses and compared their robustness with iEEG spatial sampling, introduction of spurious correlations and performance on a SOZ lateralization task. We found that (i) several methods are highly correlated and thus likely provide redundant information, and (ii) RE is more dissimilar to other connectivity methods across reference types and performs best in SOZ lateralization.

### Re-referencing methods

Re-referencing is a standard pre-processing method that aims to eliminate the influence of an imperfect reference that may be susceptible to noise. All other signals are recorded against the reference, so its influence affects every channel. CAR proposes using the mean value across all channels at a given time point as a better reference ‘electrode’ than a bone screw or a single microelectrode channel.^16^ However, the subtraction of a common reference embeds a shared signal across all channels.^33^ In the context of FC, we posited that this would result in the introduction of artificial correlations between channels. In a simulation of real data, we found that BR minimizes interchannel correlation between pairs of non-adjacent channels more than CAR. These findings are in accordance with previous work that found that BR outperformed CAR in minimizing interchannel correlation and maximizing coefficient of determination with a prescribed task.^34^ When the number of recording channels (*n*) increases, adjacent and non-adjacent pairs increase by *n* and $n^{2}$, respectively. Thus, the number of recording sites should be considered when evaluating whether to use BR or CAR; for iEEG with a large number of channels, BR may be preferred to avoid spurious correlations.

### Connectivity methods measure different properties of networks

A growing body of evidence has supported the hypothesis that epilepsy is a network disorder.^35-37^ To complement this perspective, methods from network science have been integrated with invasive EEG^2,3,5,38^ to understand properties of the epileptic network and to identify surgical targets. In this approach, network nodes represent EEG recording sites and network edges represent connectivity between pairs of recorded signals. The construction of a network from EEG data relies on selecting a re-referencing method. In a head-to-head comparison of connectivity metrics, we found that the choice of reference was more important than the choice of connectivity metric for most connectivity methods. However, RE segregated into a separate cluster, regardless of the selected re-referencing method. Based on these findings, we suggest that researchers can provide evidence of robust iEEG FC analyses by testing multiple re-referencing methods rather than performing an exhaustive sweep of different connectivity methods and frequency bands for spectral methods such as PLV and coherence. Given its distinct properties relative to other tested methods, we suggest that computing RE may be informative in exploratory analyses.

### RE outperforms other FC measures in multiple quality tests

RE is a recently described connectivity metric that was found to localize the epileptogenic zone in its original publication.^17^ We found that RE was less similar to other connectivity measures, regardless of the re-referencing method chosen. RE outperformed other FC measures in separating right and left-sided SOZs in temporal lobe epilepsy. After accounting for interictal epileptiform spikes, we found high concordance between average RE in a channel and the number of spikes detected within that epoch (Supplementary Fig. 7B). As higher RE suggests decreased FC, this finding indicates that the presence of spikes in a channel was associated with lower FC with other channels. This supports previous findings that controlling for spikes decreases FC-based prediction of SOZ channels.^27^ RE is calculated as the difference in the probability distributions of amplitude values in two signals, and so, unlike other connectivity measurements, the dynamic nature of the signal is not considered in this calculation. As entropy is a measure of randomness or heterogeneity, it is important to note that the interpretation of entropy-based connectivity measures diverges from that of time- and frequency-based measures: higher values of RE between two channels indicate lower FC between those channels. The novelty and clinical application of RE motivated its inclusion in our study. Other entropy measures, such as permutation and wavelet entropy, have previously been applied to identify statistical associations in EEG.^39,40^ As entropy-based methods are non-parametric, they do not assume characteristics of the signal distribution. However, entropy-based methods also do not leverage known properties of neural signals, such as the presence of canonical frequency bands at which neuronal populations preferentially fire. This difference between the calculation of RE and other time- and frequency-based FC measures may drive the dissimilarity of pre-processing pipelines. Further prospective work should be conducted to evaluate the neurophysiological basis for RE.

### Limitations and future work

We have aimed to present a rigorous evaluation of re-referencing and FC metrics, though certain limitations must be noted. First, the number and location of iEEG electrodes are solely determined by clinical need. Thus, there is tremendous heterogeneity across patients, driven by the clinical team's confidence in the pre-implant hypothesis. This limitation most affects our ability to lateralize the SOZ across patients, as equivalent sampling of bilateral structures is necessary. To mitigate this limitation, we focus on the subset of patients who are implanted with lateral depth electrodes bilaterally and only consider channels that have a matched, contralateral source. Though this choice of spatial sampling limits our coverage of other cortical structures, complementary channels allow us to evaluate laterality without imbalanced hemispheric coverage. The limited coverage of cortical structures with equivalent bilateral sampling hinders our ability to detect abnormalities outside of the mesial temporal lobe. Our analyses are based on 24 connectivity methods and 2 re-referencing methods, and there are many other viable methods to develop FC matrices with iEEG. We did not consider directed measures of connectivity such as partial directed coherence^41^ and Granger causality.^42^ It is also important to note that the findings of this study may not translate directly to studies in other neuropsychiatric disorders. However, our study suggests that the choice of network model and referencing technique may be important in other domains. As quantitative methods approach clinical translation, we must be rigorous and exhaustive with interpretations of pathological brain data.^31^ We have provided tools for rapidly assessing these effects that can be applied to electrophysiological data from other studies that may help provide insights into these effects. To facilitate the extension of this analysis to new methods, we publish all code and an interactive interface to build and evaluate other pre-processing pipelines on new data.

## Conclusion

This study aimed to evaluate the effect of re-referencing and connectivity methods on iEEG FC networks within the context of method similarity, reliability to incomplete spatial sampling, robustness to spurious correlations and a clinical task: the ability to lateralize the SOZ. We also developed interactive interfaces in MATLAB and Python to extend this study to new methods that we hope will be a resource to the neuroengineering community. Future research building upon these findings and exploring a wider range of pre-processing methods is essential for gaining a more comprehensive understanding of intracranial neurophysiology and improving the reproducibility of rigorous research into brain network disorders.

## Supplementary Material

fcae165_Supplementary_Data

## Data Availability

All code is available on Github at https://github.com/penn-cnt/CNT_Preprocessing_Toolkit. The analysis pipeline for reproducing all figures is given: https://github.com/penn-cnt/CNT_Preprocessing_Toolkit/blob/main/demo/ana_pipeline.m. A Readme document outlines necessary packages and instructions for replicating our methods. Both data sets are available on iEEG.org where users can request free accounts and access to the HUP_Intracranial_Data projects. Functional connectivity networks are available at https://doi.org/10.26275/4P8L-N5KT.
