# Supplementary material for: Utility of intracranial EEG networks depends on re-referencing and connectivity choice
Source: Brain Commun. 2024 May 13;6(3):fcae165. doi: 10.1093/braincomms/fcae165 (PMC11126314; doi:10.1093/braincomms/fcae165)
Supplement: fcae165_Supplementary_Data [file fcae165_supplementary_data.docx]

# Materials and Methods

**iEEG recording**

Intracranial electrode configurations (Ad Tech Medical Instruments, Racine, WI) consisted of linear depth electrodes (1.1 mm diameter with 5 mm inter-contact spacing), and linear cortical strips and two-dimensional cortical grid arrays (2.3 mm diameter with 10 mm inter-contact spacing) for the HUP cohort. For the MUSC cohort, electrode configurations consisted of linear depth electrodes (0.86mm diameter with 5 mm inter-contact spacing). Recording sampling rates varied from 256-1024 Hz (Table 1). All signals were referenced to an electrode distant from the suspected seizure generators, usually embedded in medullary bone in the skull.

**Electrode localization**

Pre- and post-implant T1-weighted MPRAGE MRIs, and post-implant CT images were obtained for localization of electrodes. iEEG-recon was used to digitize electrode coordinates on CT scans. Pre- and post-implant images were linearly registered and in-house software was used for electrode localization^1^. The Desikan-Killiany-Tourville atlas in ANTsPyNet was used to assign regions of interest in each electrode and Atropos-style six tissue segmentation was performed to assign contacts to gray and white matter^2,3^.

**Seizure localization and surgical outcome**

Seizures, seizure-onset electrodes, and the anatomical seizure onset localization were initially identified by a board-certified epileptologist for clinical purposes, and further confirmed in an epilepsy clinical case conference. Seizures agreed by clinicians were included, regardless of onset pattern, location, or semiology. Seizure onset electrodes were first identified as those showing the earliest electrographic changes. Seizure onset zones and seizure lateralization were then determined, with mesial temporal and temporal neocortical anatomical seizure localizations classified as “temporal”, and all other localizations, including those that were diffuse and multifocal (including patients who had multiple seizures coming from different locations), classified as “non-temporal”.

## Robustness of clustering agreement between HUP and MUSC cohorts

To determine if our results were consistent on an external validation set, we tested multiple statistics for the similarity of cluster assignments. First, we computed FC networks using each of the 48 preprocessing pipelines. We then computed pairwise Pearson correlation between the outputs of each preprocessing pipeline within each patient, and then averaged across patients within one epilepsy center. Thus, each of the 48 preprocessing pipelines had a Pearson correlation value with every other preprocessing pipeline. On each 48x48 correlation matrix, we tested 9 clustering algorithms: 1) Louvain community detection, 2) K-means clustering, 3) spectral clustering, 4) agglomerative clustering, 5) affinity propagation, 6) Birch clustering, 7) DBSCAN clustering, 8) mean shift clustering, and 9) OPTICS clustering. We chose a suite of clustering algorithms to overcome the limitations of any single clustering algorithm. Each clustering algorithm was applied for 100 iterations and condensed into one clustering assignment using a consensus partition with the reclustering resolution threshold τ = 0.1. To compare cluster assignments between HUP and MUSC, we applied 7 clustering similarity metrics: 1) adjusted Rand index, 2) adjusted mutual information, 3) normalized mutual information, 4) Fowlkes Mallow score, 5) homogeneity, 6) completeness, and 7) V measure. All 7 metrics are measures from 0 to 1 and visualized in Supplementary Figure 1. Most clustering algorithms and cluster similarity metrics were implemented in sklearn.cluster and sklearn.metrics, respectively. Louvain community detection and consensus clustering were implemented in the Brain Connectivity Toolbox for Python.

# Results

**Supplementary Table 1.** **Methods Description**

| **Method Category** | **Method Name** | **Description** | **Formula** |
| --- | --- | --- | --- |
| Re-referencing  Methods | Common Average Re-referencing | Subtracting the average signal across all electrodes from each electrode's signal to remove common noise from recorded signals. | $x_{i}' =x_{i} - \underline{x}$ |
|  | Bipolar Re-referencing | Subtracting the signal of the consecutive contact on the same electrode from each signal. Output signal being the difference between neighboring electrodes. | $x_{i}' = x_{i} - x_{i+1}$ |
| Connectivity Methods | Pearson Correlation | A statistical measure that quantifies the linear relationship between two variables. It calculates a correlation coefficient, ranging from -1 to 1, which represents the strength and direction of the association. A coefficient of 1 indicates a perfect positive correlation, -1 represents a perfect negative correlation, and 0 indicates no linear correlation between the variables. | 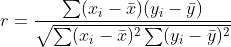 |
|  | Squared Pearson Correlation | This method squares the correlation coefficient of Pearson Correlation, and represents the fraction of the variation in one variable that may be explained by the other variable. It ranges from 0 to 1, where 0 indicates no shared variance and 1 represents complete shared variance. | $r^{2}$ |
|  | Cross Correlation | Measure the similarity between two signals as one signal is shifted in time relative to the other. It calculates the degree of similarity at the time lag at which the signals are most aligned. | 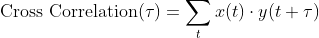 |
|  | Coherence | Also called magnitude-squared coherence. A measure of the linear relationship between two signals in the frequency domain. Calculated as the squared magnitude of the cross-spectral density (CSD) divided by the product of the power spectral densities (PSD) of the two signals. Range from 0 to 1. | 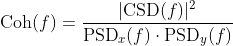 |
|  | Phase Locking Value | A measure of phase synchronization between two signals. It quantifies the level of synchronization between certain oscillatory components of two signals. It computes the mean phase difference between the two signals and expresses it as a complex unit-length vector. The absolute value is used as PLV, which ranges from 0 to 1. | 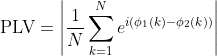 |
|  | Relative Entropy | Also known as Kullback-Leibler (KL) divergence. A measure of the dissimilarity between two probability distributions. The probability distributions are defined to be the distribution in amplitudes of the EEG signal. A value of 0 indicates perfect similarity. | 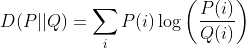 |

**Supplementary Table 2. Statistical Results for Connectivity Measure Reliability Pairwise Comparison for the HUP dataset**

|  | Pearson | Squared Pearson | Cross Correlation | Coherence | PLV | Relative Entropy |
| --- | --- | --- | --- | --- | --- | --- |
| Lower 20%, Upper 40% | | | | | | |
| Pearson | / | -3.50**** | -1.68**** | -1.33**** | -2.38**** | -4.78**** |
| Squared Pearson | -3.49**** | / | 1.82**** | 2.17**** | 1.12*** | -1.28**** |
| Cross Correlation | -1.83**** | 1.67**** | / | 0.36 (n.s.) | -0.70 (n.s.) | -3.09**** |
| Coherence | -1.25**** | 2.24**** | 0.58 (n.s.) | / | -1.06*** | -3.45**** |
| PLV | -2.18**** | 1.31**** | -0.36 (n.s.) | -0.93** | / | -2.40**** |
| Relative Entropy | -4.75**** | -1.26**** | -2.92**** | -3.50**** | -2.57**** | / |
| Lower 60%, Upper 80% | | | | | | |
| Pearson | / | -3.01**** | -1.27**** | -1.61**** | -3.05**** | -4.79**** |
| Squared Pearson | -3.28**** | / | 1.74**** | 1.40**** | -0.04 (n.s.) | -1.78**** |
| Cross Correlation | -1.48**** | 1.81**** | / | -0.34 (n.s.) | -1.78**** | -3.52**** |
| Coherence | -1.41**** | 1.87**** | 0.07 (n.s.) | / | -1.44**** | -3.18**** |
| PLV | -2.72**** | 0.56 (n.s.) | -1.25**** | -1.31**** | / | -1.74**** |
| Relative Entropy | -4.78**** | -1.50**** | -3.30**** | -3.37**** | -2.06**** | / |

Notes. Cells report pairwise comparison mean rank differences and significance from post hoc Dunn-Šidák multiple comparisons tests following Friedman test. Lower and upper triangles represent significance values at different spatial sample percentages. For lower triangles, cells report differences of the measure in columns minus that in rows; for upper triangles, cells report differences of the measure in rows minus that in rows. ****, p < 0.0001; ***, p < 0.001; **, p < 0.01; *, p < 0.05, n.s., p >= 0.05.

**Supplementary Table 3. Statistical Results for Frequency Bands Pairwise Comparison for the HUP dataset**

|  | Delta | Theta | Alpha | Beta | Gamma | Ripple | Broad |
| --- | --- | --- | --- | --- | --- | --- | --- |
| Lower 20%, Upper 40% | | | | | | | |
| Delta | / | 1.01* | 1.79**** | 3.27**** | 4.75**** | 4.55**** | 2.59**** |
| Theta | 0.98* | / | 0.78 (n.s.) | 2.26**** | 3.74**** | 3.54**** | 1.58**** |
| Alpha | 1.63**** | 0.65 (n.s.) | / | 1.48**** | 2.95**** | 2.75**** | 0.80 (n.s.) |
| Beta | 3.03**** | 2.05**** | 1.40**** | / | 1.47**** | 1.27*** | -0.68 (n.s.) |
| Gamma | 4.58**** | 3.60**** | 2.95**** | 1.56**** | / | -0.20 (n.s.) | -2.15**** |
| Ripple | 4.80**** | 3.82**** | 3.17**** | 1.77**** | 0.22 (n.s.) | / | -1.95**** |
| Broad | 3.07**** | 2.08**** | 1.43**** | 0.04 (n.s.) | -1.52**** | -1.74**** | / |
| Lower 60%, Upper 80% | | | | | | | |
| Delta | / | 1.12** | 2.13**** | 3.25**** | 3.98**** | 2.35**** | 0.24 (n.s.) |
| Theta | 1.09** | / | 1.01* | 2.13**** | 2.86**** | 1.23*** | -0.89 (n.s.) |
| Alpha | 1.98**** | 0.89 (n.s.) | / | 1.12** | 1.85**** | 0.22 (n.s.) | -1.90**** |
| Beta | 3.51**** | 2.42**** | 1.53**** | / | 0.73 (n.s.) | -0.91* | -3.02**** |
| Gamma | 4.67**** | 3.58**** | 2.69**** | 1.16** | / | -1.63**** | -3.75**** |
| Ripple | 4.03**** | 2.93**** | 2.05**** | 0.52 (n.s.) | -0.64 (n.s.) | / | -2.11**** |
| Broad | 1.89**** | 0.79 (n.s.) | -0.09 (n.s.) | -1.62**** | -2.78**** | -2.14**** | / |

Notes. Cells report pairwise comparison mean rank differences significance from post hoc Dunn-Šidák multiple comparisons tests following Friedman test. Lower and upper triangles represent significance values at different spatial sample percentages. For lower triangles, cells report differences of the measure in columns minus that in rows; for upper triangles, cells report differences of the measure in rows minus that in rows. ****, p < 0.0001; ***, p < 0.001; **, p < 0.01; *, p < 0.05, n.s., p >= 0.05.

**Supplementary Table 4. Statistical results for SOZ versus non-SOZ side global connectivity comparison**

|  | HUP dataset | | | | MUSC dataset | | | |
| --- | --- | --- | --- | --- | --- | --- | --- | --- |
|  | Percent  Change % | *t*-statistics | BH-corrected *p*-value | Cohen’s d | Percent  Change % | *t*-statistics | BH-corrected *p*-value | Cohen’s d |
| CAR-Pearson | 0.118 | -0.764 | 0.759 | -0.142 | -0.230 | -2.955 | 0.085 | -0.763 |
| CAR-SquareP | 0.155 | 0.394 | 0.898 | 0.073 | -0.230 | -2.955 | 0.085 | -0.763 |
| CAR-CrossCorr | -0.477 | -1.396 | 0.460 | -0.259 | -0.070 | -3.028 | 0.085 | -0.782 |
| CAR-COH-Delta | 0.002 | -0.208 | 0.940 | -0.039 | -0.024 | -2.613 | 0.104 | -0.675 |
| CAR-COH-Theta | 0.005 | -0.103 | 0.966 | -0.019 | -0.023 | -1.547 | 0.342 | -0.399 |
| CAR-COH-Alpha | 0.005 | -0.092 | 0.966 | -0.017 | -0.013 | -1.133 | 0.508 | -0.292 |
| CAR-COH-Beta | -0.006 | -0.648 | 0.759 | -0.120 | 0.008 | 0.768 | 0.682 | 0.198 |
| CAR-COH-Gamma | -0.006 | -0.598 | 0.783 | -0.111 | 0.003 | 0.170 | 0.915 | 0.044 |
| CAR-COH-Ripple | -0.008 | -0.727 | 0.759 | -0.135 | 0.003 | 0.247 | 0.912 | 0.064 |
| CAR-COH-Broad | -0.006 | -0.661 | 0.759 | -0.123 | 0.001 | 0.158 | 0.915 | 0.041 |
| CAR-PLV-Delta | 0.007 | -0.068 | 0.966 | -0.013 | -0.027 | -2.405 | 0.129 | -0.621 |
| CAR-PLV-Theta | 0.001 | -0.339 | 0.898 | -0.063 | -0.026 | -1.312 | 0.444 | -0.339 |
| CAR-PLV-Alpha | 0.005 | -0.201 | 0.940 | -0.037 | -0.014 | -0.967 | 0.558 | -0.250 |
| CAR-PLV-Beta | -0.002 | -0.680 | 0.759 | -0.126 | 0.005 | 0.091 | 0.929 | 0.024 |
| CAR-PLV-Gamma | -0.002 | -0.792 | 0.759 | -0.147 | 0.020 | 0.236 | 0.912 | 0.061 |
| CAR-PLV-Ripple | 0.043 | -0.000 | 1.000 | -0.000 | 0.061 | 1.040 | 0.540 | 0.268 |
| CAR-PLV-Broad | -0.000 | -1.062 | 0.570 | -0.197 | -0.013 | -0.733 | 0.684 | -0.189 |
| CAR-RE-Delta | 0.103 | 2.406 | 0.099 | 0.447 | 0.046 | 1.607 | 0.326 | 0.415 |
| CAR-RE-Theta | 0.104 | 3.220 | 0.019 | 0.598 | 0.059 | 1.210 | 0.478 | 0.312 |
| CAR-RE-Alpha | 0.118 | 3.614 | 0.018 | 0.671 | 0.089 | 1.406 | 0.412 | 0.363 |
| CAR-RE-Beta | 0.503 | 4.083 | 0.015 | 0.758 | 0.371 | 2.248 | 0.160 | 0.580 |
| CAR-RE-Gamma | 0.710 | 3.264 | 0.019 | 0.606 | 0.451 | 2.041 | 0.180 | 0.527 |
| CAR-RE-Ripple | 0.448 | 2.110 | 0.146 | 0.392 | 0.424 | 1.761 | 0.263 | 0.455 |
| CAR-RE-Broad | 0.311 | 3.513 | 0.018 | 0.652 | 0.180 | 2.138 | 0.173 | 0.552 |
| BR-Pearson | -1.065 | -1.208 | 0.517 | -0.224 | -3.172 | 0.535 | 0.739 | 0.138 |
| BR-SquareP | 0.006 | -1.337 | 0.460 | -0.248 | -3.172 | 0.535 | 0.739 | 0.138 |
| BR-CrossCorr | 0.163 | -1.270 | 0.490 | -0.236 | 0.055 | 1.077 | 0.531 | 0.278 |
| BR-COH-Delta | -0.007 | -0.668 | 0.759 | -0.124 | 0.021 | 1.199 | 0.478 | 0.310 |
| BR-COH-Theta | -0.003 | -0.475 | 0.876 | -0.088 | 0.010 | 0.440 | 0.799 | 0.114 |
| BR-COH-Alpha | 0.007 | 0.298 | 0.899 | 0.055 | 0.004 | 0.093 | 0.929 | 0.024 |
| BR-COH-Beta | 0.024 | 1.604 | 0.359 | 0.298 | 0.009 | 0.878 | 0.610 | 0.227 |
| BR-COH-Gamma | 0.018 | 1.515 | 0.397 | 0.281 | 0.009 | 2.948 | 0.085 | 0.761 |
| BR-COH-Ripple | 0.010 | 0.870 | 0.723 | 0.162 | 0.004 | 3.110 | 0.085 | 0.803 |
| BR-COH-Broad | 0.012 | 1.140 | 0.550 | 0.212 | 0.006 | 2.917 | 0.085 | 0.753 |
| BR-PLV-Delta | -0.034 | -2.089 | 0.146 | -0.388 | 0.022 | 1.301 | 0.444 | 0.336 |
| BR-PLV-Theta | -0.022 | -1.342 | 0.460 | -0.249 | 0.014 | 0.641 | 0.719 | 0.166 |
| BR-PLV-Alpha | -0.002 | -0.394 | 0.898 | -0.073 | 0.000 | -0.177 | 0.915 | -0.046 |
| BR-PLV-Beta | 0.001 | -0.329 | 0.898 | -0.061 | -0.009 | -0.628 | 0.719 | -0.162 |
| BR-PLV-Gamma | 0.035 | 0.324 | 0.898 | 0.060 | 0.013 | 0.985 | 0.558 | 0.254 |
| BR-PLV-Ripple | 0.013 | 0.092 | 0.966 | 0.017 | 0.018 | 0.412 | 0.803 | 0.106 |
| BR-PLV-Broad | -0.010 | -1.105 | 0.557 | -0.205 | 0.058 | 2.636 | 0.104 | 0.681 |
| BR-RE-Delta | 0.088 | 2.248 | 0.121 | 0.417 | 0.019 | 0.590 | 0.732 | 0.152 |
| BR-RE-Theta | 0.096 | 2.739 | 0.056 | 0.509 | 0.037 | 0.717 | 0.684 | 0.185 |
| BR-RE-Alpha | 0.115 | 3.224 | 0.019 | 0.599 | 0.089 | 1.782 | 0.263 | 0.460 |
| BR-RE-Beta | 0.482 | 3.810 | 0.016 | 0.708 | 0.413 | 2.421 | 0.129 | 0.625 |
| BR-RE-Gamma | 0.678 | 3.406 | 0.019 | 0.632 | 0.582 | 2.127 | 0.173 | 0.549 |
| BR-RE-Ripple | 0.364 | 2.243 | 0.121 | 0.416 | 0.367 | 2.034 | 0.180 | 0.525 |
| BR-RE-Broad | 0.308 | 2.620 | 0.066 | 0.486 | 0.182 | 2.682 | 0.104 | 0.693 |

Notes. Global connectivity percent changes calculated from (SOZ - non-SOZ) / non-SOZ. Significances in SOZ versus non-SOZ side connectivity differences were measured using a paired t-test with Benjamini-Hochberg correction. Effect sizes were estimated using Cohen’s d. CAR, common average re-referencing; BR, bipolar re-referencing; Pearson, Pearson correlation; SquareP, Squared Pearson correlation; CrossCorr, cross correlation; COH, coherence; PLV, phase-locking value; RE, relative entropy.


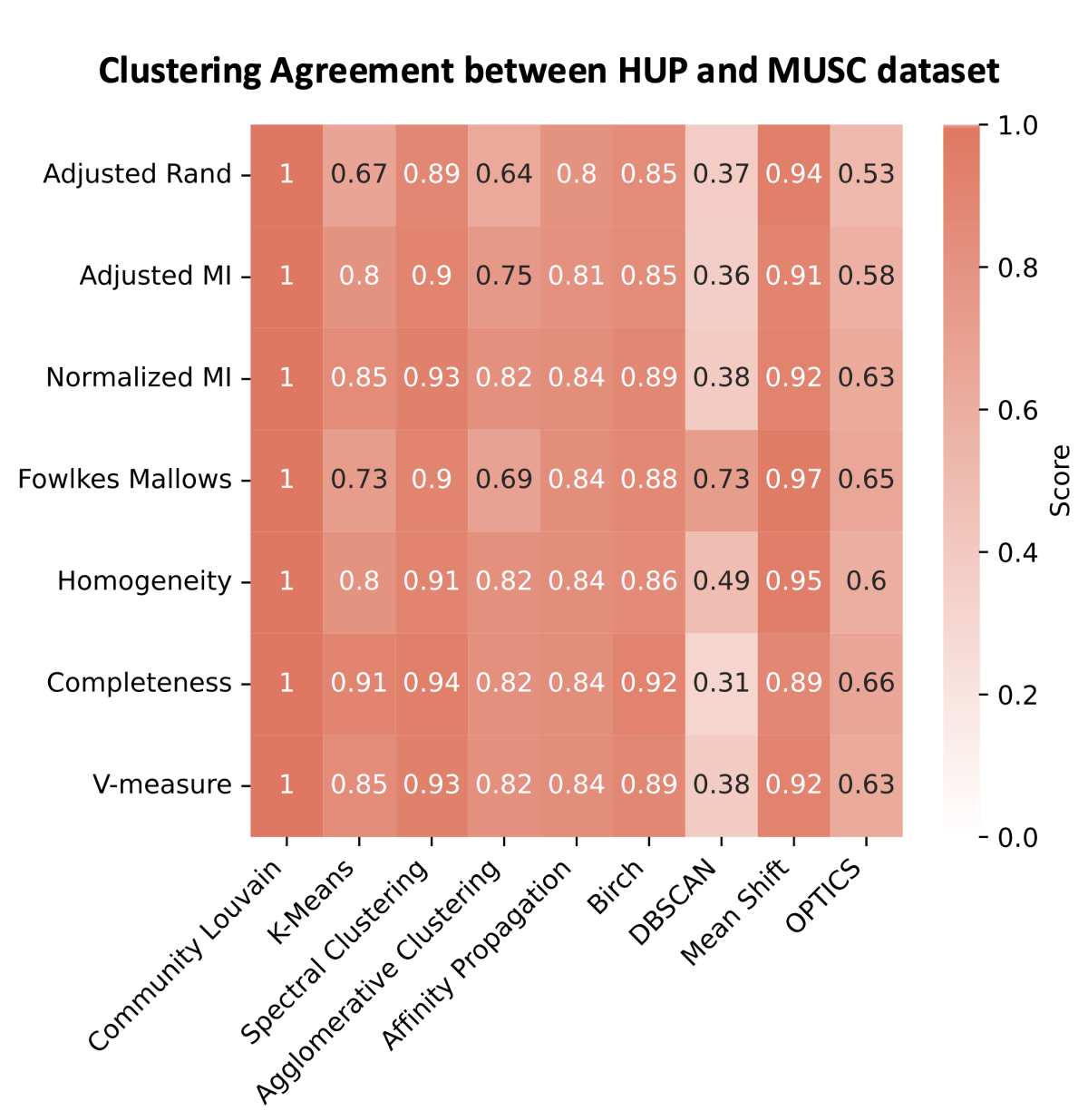


**Supplementary Figure 1. Clustering agreement between cohorts.** We assessed the robustness of clustering and clustering agreement between Hospital of the University of Pennsylvania (HUP, *n* = 107 subjects) and Medical University of South Carolina (MUSC, *n* = 18 subjects) cohorts by applying 9 clustering algorithms and 7 cluster similarity metrics. Clustering algorithms applied were 1) Louvain community detection, 2) K-means clustering, 3) spectral clustering, 4) agglomerative clustering, 5) affinity propagation, 6) Birch clustering, 7) Density-Based Spatial Clustering of Applications with Noise (DBSCAN) clustering, 8) mean shift clustering, and 9) Ordering points to identify the clustering structure (OPTICS) clustering. To compare cluster assignments between HUP and MUSC, we applied 7 clustering similarity metrics: 1) adjusted Rand index, 2) adjusted mutual information (MI), 3) normalized mutual information, 4) Fowlkes Mallow score, 5) homogeneity, 6) completeness, and 7) V measure. Each cell of the heatmap represents a statistical test (row) applied to a clustering algorithm (column), all statistical tests are bounded from 0 to 1.


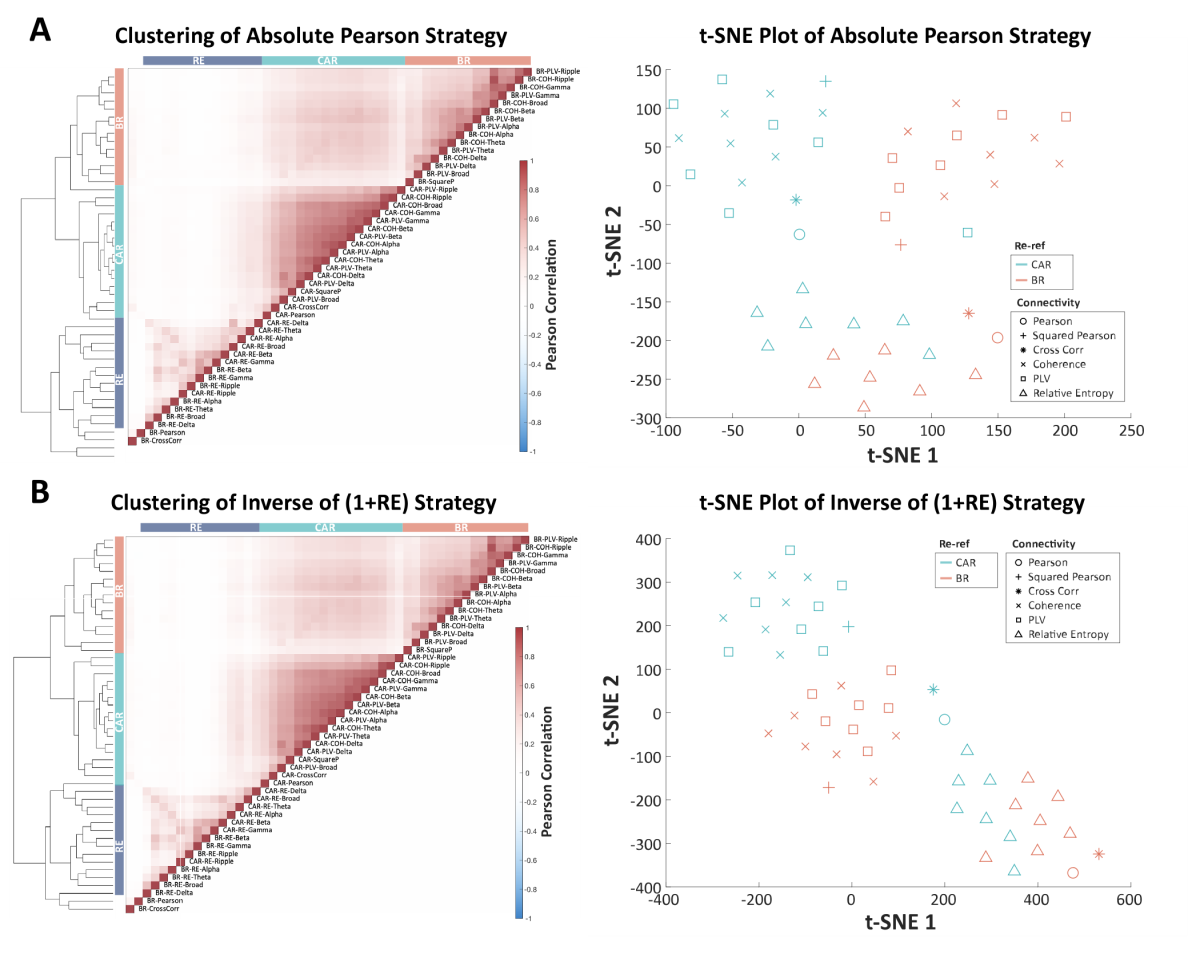


**Supplementary Figure 2. Additional examination of preprocessing pipeline clustering.** To avoid methodology bias in the clustering of preprocessing pipelines, A) the absolute value of the signed Pearson similarity matrix was taken, B) the connectivity of pipelines involving relative entropy were transformed through taking the inverse of 1+connectivity to accommodate the differed range of relative entropy (RE, 0 - infinite). Left, hierarchical clustering of pipelines, each row and column represents a pre-processing pipeline, colors along the axes represent three major clusters: CAR = common average re-referencing cluster, BR = bipolar re-referencing cluster, RE = relative entropy cluster. Cell colors show the Pearson correlation coefficient between pairs of preprocessing pipelines. Right, t-SNE (t-distributed stochastic neighbor embedding) plot of the similarity matrix. Three clusters emerged in both cases (A: average stability = 0.95, B: average stability = 0.93).


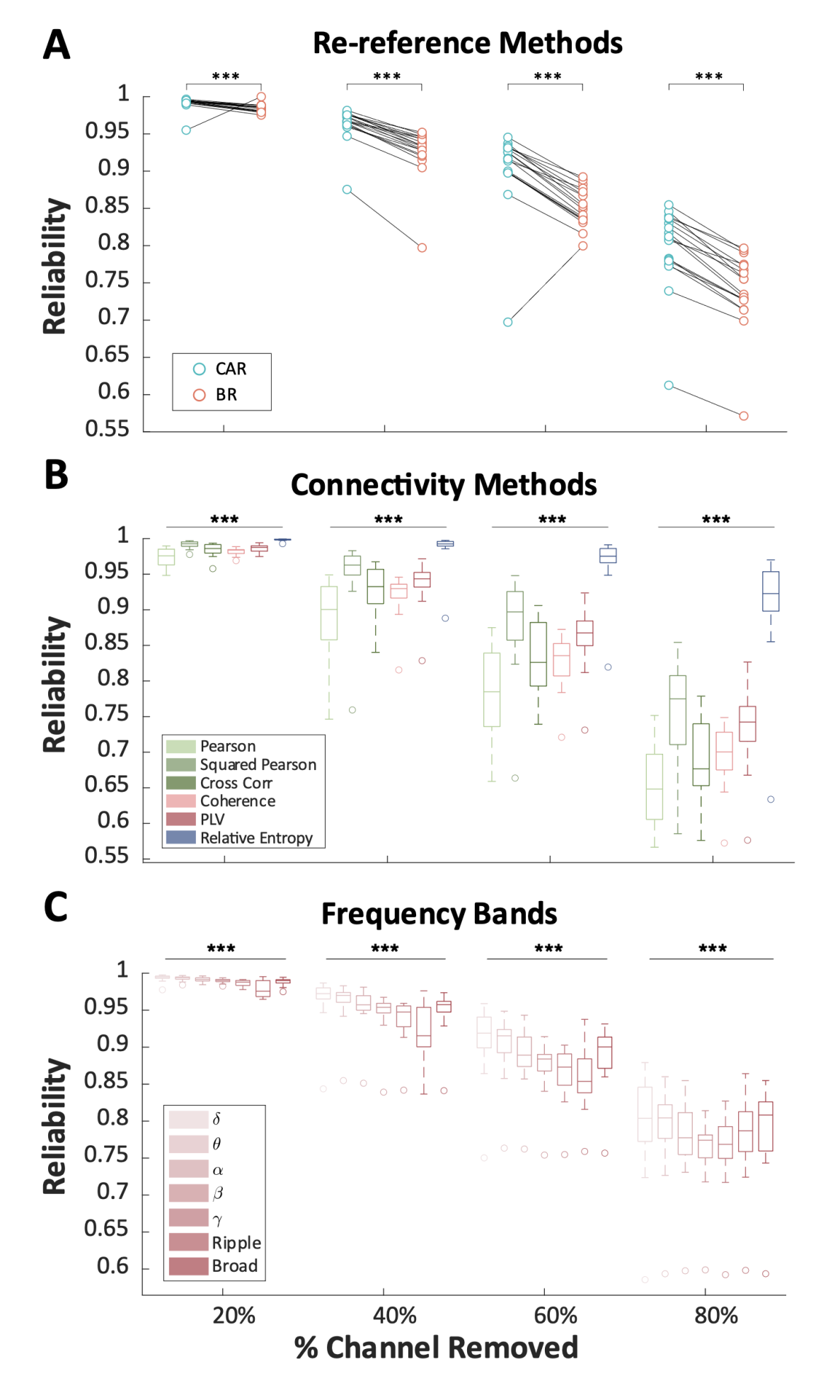


**Supplementary Figure 3. Robustness of spatial sampling of different preprocessing pipelines in the Medical University of South Carolina (MUSC) dataset.** A) The Reliability of different pipelines involving the focused re-referencing method were averaged. Each point represents a patient. A Wilcoxon signed rank test was applied. 20%: Wilcoxon signed rank test Z = 2.94, p = 0.003, 40%: Z = 3.72, p < 0.001, 60%: Z = 2.94, p = 0.003, 80%: Z = 3.72, p < 0.001. B) Average reliability of the connectivity method across different re-referencing methods was reported. Non-parametric Friedman tests were used to examine differences in robustness across methods. 20%: Friedman test χ^2^ = 67.75, p < 0.001, 40%: χ^2^ = 65.46, p < 0.001, 60%: χ^2^ = 67.33, p < 0.001, 80%: χ^2^ = 74.54, p < 0.001. Post hoc Dunn-Šidák multiple comparisons tests were further conducted for the Friedman test to identify significantly different method pairs. C) Boxplots group each spectral connectivity method by the canonical frequency band that was used. Friedman test and multiple comparisons corrections were applied. 20%: Friedman test χ^2^ = 64.07, p < 0.001, 40%: χ^2^ = 63.02, p < 0.001, 60%: χ^2^ = 48.98, p < 0.001, 80%: χ^2^ = 34.29, p < 0.001. CAR = common average re-referencing, BR = bipolar re-referencing, RE = relative entropy, Pearson = Pearson correlation, CrossCorr = cross-correlation, SquareP = squared Pearson correlation, PLV = phase locking value, COH = magnitude-squared coherence. Greek letters refer to canonical frequency bands.


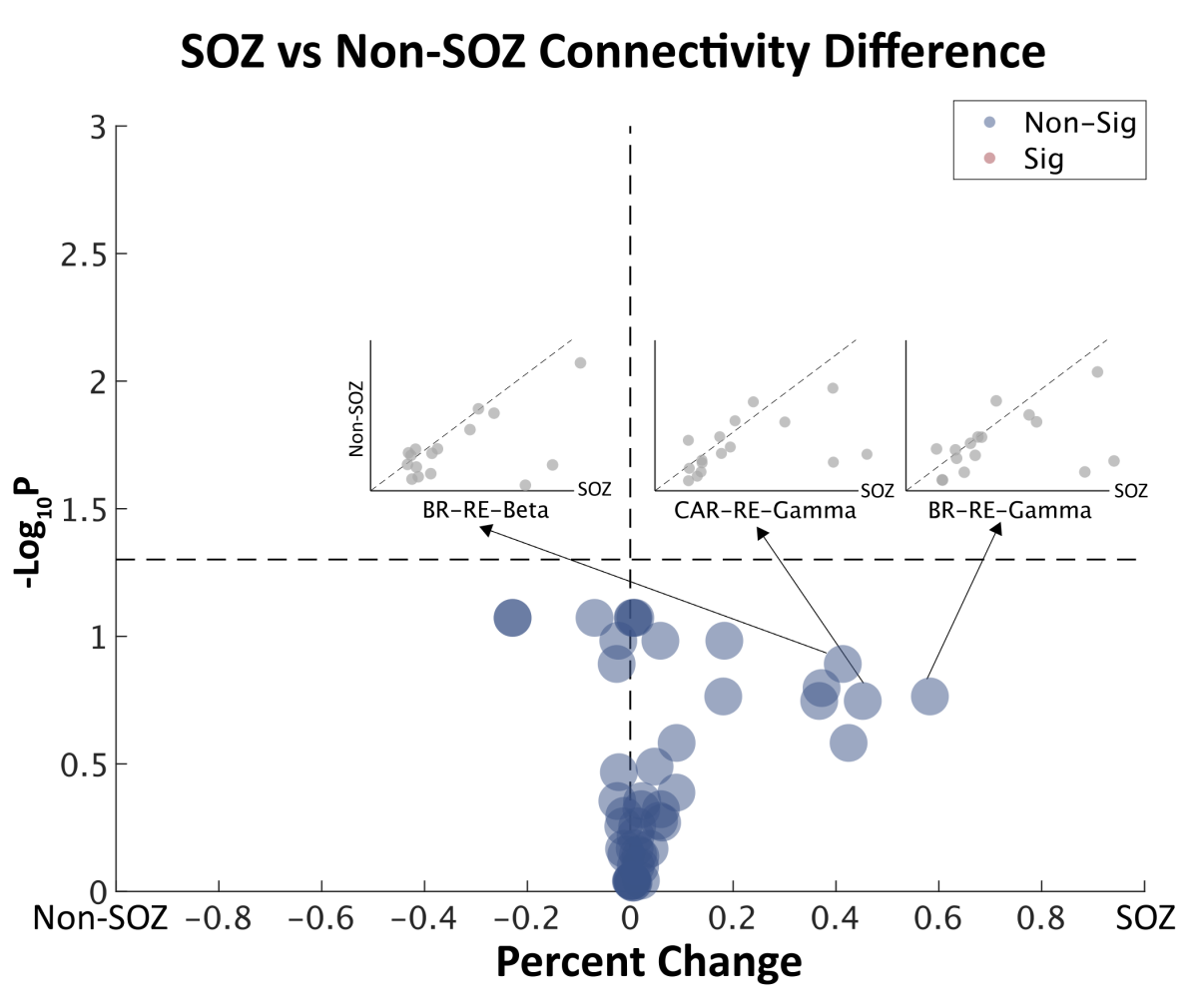


**Supplementary Figure 4.** **SOZ lateralization performances of different preprocessing pipelines in the Medical University of South Carolina (MUSC) dataset. Outer:** Seizure onset zone (SOZ) versus non-seizure onset zone (non-SOZ) side global connectivity differences. Horizontal axis represents SOZ v.s. non-SOZ global connectivity change. Vertical axis indicates Benjamini Hochberg-corrected paired t-test significance of SOZ v.s. non-SOZ differences. Specifically, global connectivity was calculated through averaging connectivity of all channel pairs within each hemisphere. For each preprocessing pipeline, a paired t-test was performed to compare the global connectivity from the SOZ side v.s. that from the non-SOZ side. Each dot represents a preprocessing pipeline. **Inner:** Embedded are the sample scatter plots of SOZ versus non-SOZ side global connectivity for pipelines showing marked differences. (BR-RE-Beta, %change = 0.413, t = 2.421, p = 0.129; CAR-RE-Gamma, %change = 0.451, t = 2.041, p = 0.180; BR-RE-Gamma, %change = 0.582, t = 2.127, p = 0.173). The *p*-values and corresponding *t*-statistics of other pipelines were available in Supplementary Table 4. The horizontal and vertical axes represent the SOZ and non-SOZ side global connectivity, respectively. The dashed line indicates equal connectivity at both sides. CAR = common average re-referencing, BR = bipolar re-referencing, RE = relative entropy, Sig = statistically significant following multiple comparisons correction.


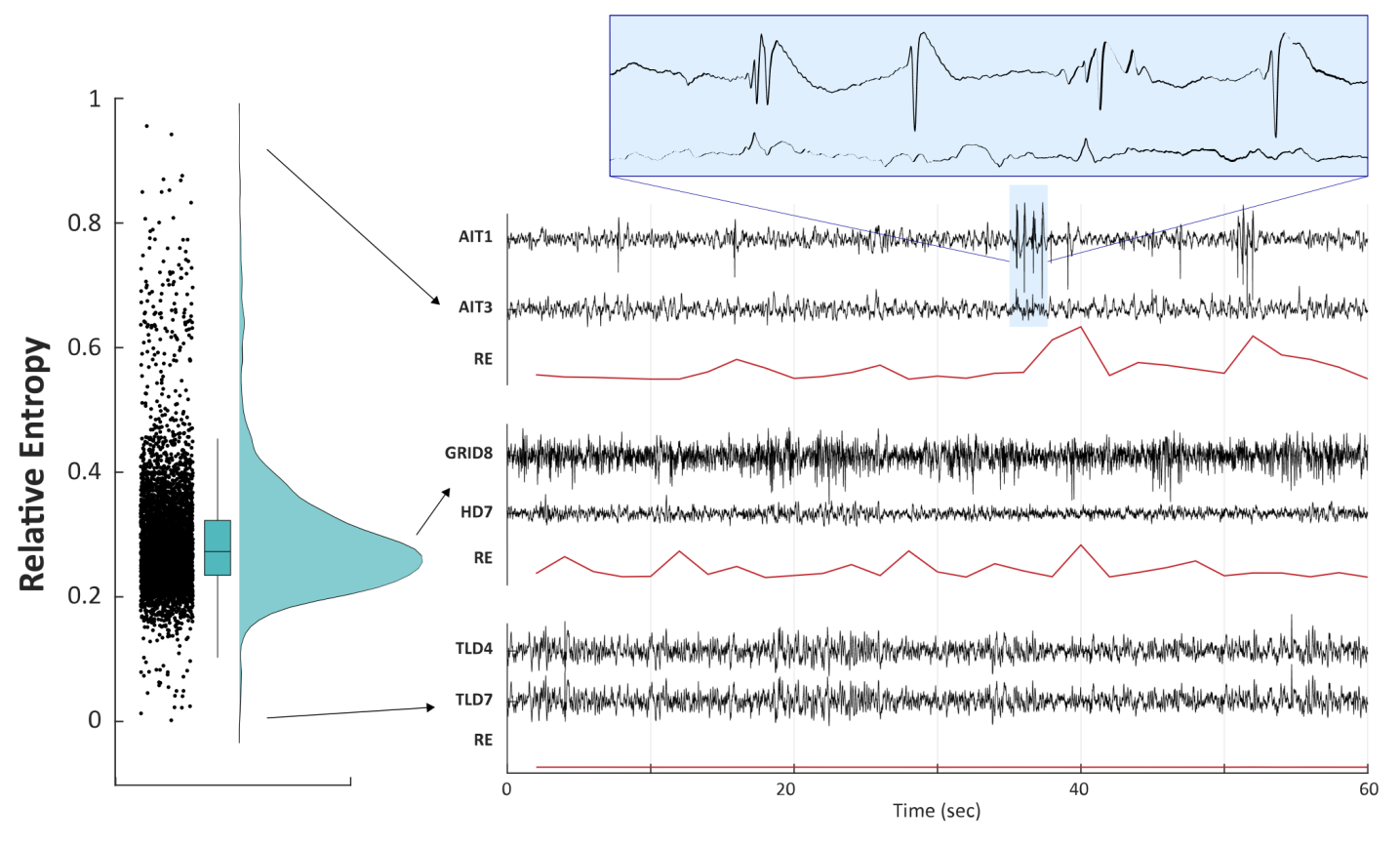


**Supplementary Figure 5. Sample intracranial EEG traces at different relative entropy levels.**

Left: Distribution of relative entropy connectivity for all channel pairs from a sample patient. Right: Sample traces of intracranial EEG (iEEG) channel pairs with high (top), intermediate (middle), and low (bottom) relative entropy (RE). The red curve represents the broadband relative entropy time series of the channel pair. Values are calculated in 2-second non-overlapping windows. Zoom-in view of the top iEEG traces highlights potential spikes that RE captures.


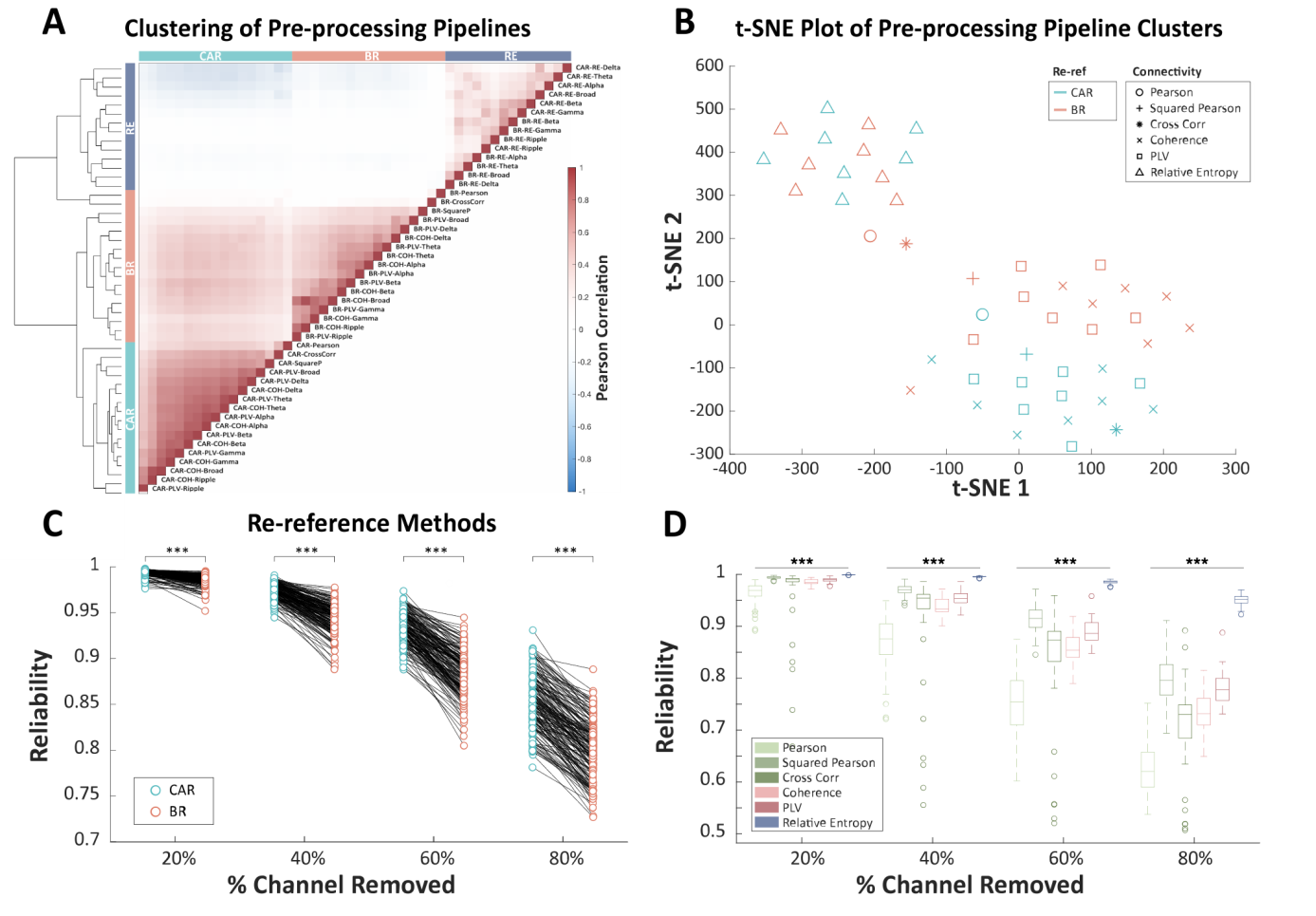


**Supplementary Figure 6. Replication of analyses in subsample of patients with at least 92 channels.** Patients from the Hospital of the University of Pennsylvania (HUP) and the Medical University of South Carolina (MUSC) datasets were combined (n = 125), and those with less than 92 channels were excluded (n = 60). Clustering and robustness of spatial sampling of different preprocessing pipelines were replicated. (A) Each row and column represents a pre-processing pipeline, colors along the axes represent three major clusters: common average re-referencing cluster, bipolar re-referencing cluster, and relative entropy cluster. Hierarchical clustering was applied to the networks and the resulting dendrogram is shown on the left axis. Cell colors show the Pearson correlation coefficient between pairs of preprocessing pipelines. Preprocessing pipelines are indicated as [Re-referencing method]-[Connectivity method]-[Frequency band (where applicable)]. (B) The similarity matrix was further reduced to 2-dimensions using t-distributed stochastic neighbor embedding (t-SNE). Each data point refers to a pre-processing pipeline. (C) The reliability of different pipelines involving the focused re-referencing method were averaged. Each point represents a patient. A Wilcoxon signed rank test was applied. All percentages, p < 0.001. (D) Average reliability of the connectivity method across different re-referencing methods was reported. Non-parametric Friedman tests were used to examine differences in robustness across methods. 20%: Friedman test χ^2^ = 262.92, p < 0.001, 40%: χ^2^ = 266.63, p < 0.001, 60%: χ^2^ = 269.02, p < 0.001, 80%: χ^2^ = 274.18, p < 0.001. CAR = common average re-referencing, BR = bipolar re-referencing, RE = relative entropy, Pearson = Pearson correlation, CrossCorr = cross-correlation, SquareP = squared Pearson correlation, PLV = phase locking value, COH = magnitude-squared coherence.


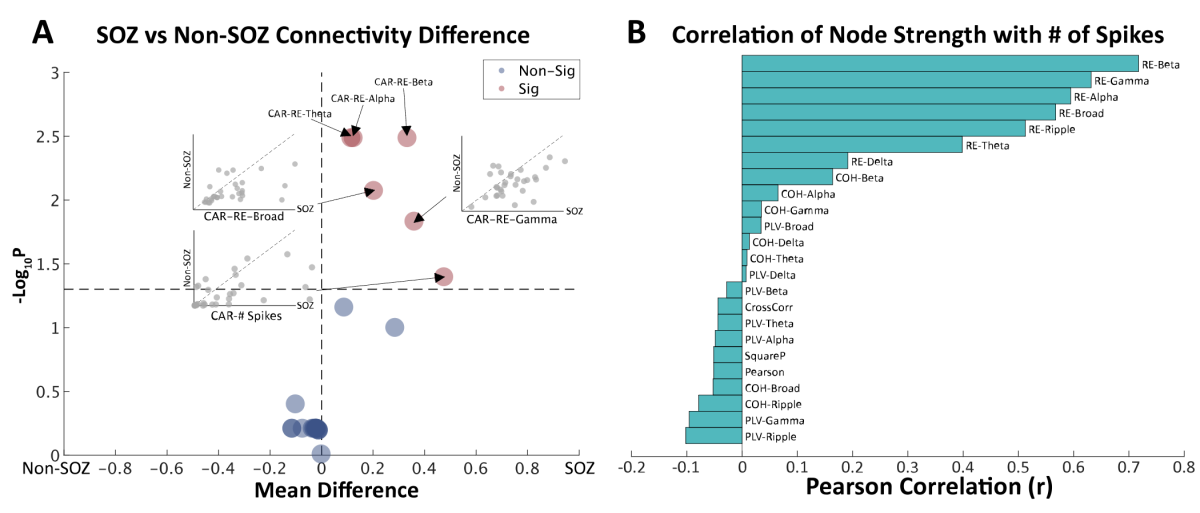


**Supplementary Figure 7. Comparison of connectivity measures with # of spikes.** (A) SOZ lateralization performances of different connectivity measures and # of spikes in the Hospital of the University of Pennsylvania (HUP) dataset. **Outer:** Seizure onset zone (SOZ) versus non-seizure onset zone (non-SOZ) side global connectivity or spike differences. Horizontal axis represents SOZ v.s. non-SOZ global connectivity difference. Vertical axis indicates Benjamini Hochberg-corrected paired t-test significance of SOZ v.s. non-SOZ differences. For each method, a paired t-test was performed to compare the global connectivity from the SOZ side v.s. that from the non-SOZ side. Each dot represents a connectivity method, with common average re-referencing performed. **Inner:** Embedded are the sample scatter plots of SOZ versus non-SOZ side global connectivity for methods showing significant differences. The horizontal and vertical axes represent the SOZ and non-SOZ side global connectivity/# of spikes, respectively. The dashed line indicates equal values at both sides. (B) Pearson correlation of channel node strength with number of spikes detected. Bar represents correlation coefficient. Common average re-referencing performed before connectivity calculation. Node strength calculated through averaging its connectivity with all other channels. CAR = common average re-referencing, BR = bipolar re-referencing, RE = relative entropy, Pearson = Pearson correlation, CrossCorr = cross-correlation, SquareP = squared Pearson correlation, PLV = phase locking value, COH = magnitude-squared coherence, Sig = statistically significant following multiple comparisons correction.

**References**

1. Lucas A, Scheid BH, Pattnaik AR, et al. iEEG-recon: A fast and scalable pipeline for accurate reconstruction of intracranial electrodes and implantable devices. *Epilepsia*. 2024;65(3):817-829. doi:10.1111/epi.17863

2. Tustison NJ, Cook PA, Holbrook AJ, et al. The ANTsX ecosystem for quantitative biological and medical imaging. *Sci Rep*. 2021;11(1):9068. doi:10.1038/s41598-021-87564-6

3. Avants BB, Tustison N, Song G. Advanced normalization tools (ANTS). *Insight J*. 2009;2(365):1-35.
